# Production and Characterization of Bioplastic by Polyhydroxybutyrate Accumulating *Erythrobacter aquimaris* Isolated from Mangrove Rhizosphere

**DOI:** 10.3390/molecules25010179

**Published:** 2020-01-01

**Authors:** Yasser S. Mostafa, Sulaiman A. Alrumman, Kholod A. Otaif, Saad A. Alamri, Mohamed S. Mostafa, Taher Sahlabji

**Affiliations:** 1 Department of Biology, College of Science, King Khalid University, P.O. Box 9004, Abha 61413, Saudi Arabia; salrumman@kku.edu.sa (S.A.A.); kh.otaif1410@gmail.com (K.A.O.); amri555@yahoo.com (S.A.A.); 2 Prince Sultan Bin Abdulaziz Center for Environmental and Tourism Research and Studies, King Khalid University, P.O. Box 9004, Abha 61413, Saudi Arabia; 3 Department of Chemistry, Faculty of Science, Jazan University, P.O. Box 114, Jazan 45142, Saudi Arabia; ms-mostafa@hotmail.com; 4 Department of Chemistry, College of Science, King Khalid University, P.O. Box 9004, Abha 61413, Saudi Arabia; tsahlabji@kku.edu.sa

**Keywords:** poly-β-hydroxybutyrate, *Erythrobacter aquimaris*, 16S rRNA gene, mangrove, FTIR, NMR, GC-MS

## Abstract

The synthesis of bioplastic from marine microbes has a great attendance in the realm of biotechnological applications for sustainable eco-management. This study aims to isolate novel strains of poly-β-hydroxybutyrate (PHB)-producing bacteria from the mangrove rhizosphere, Red Sea, Saudi Arabia, and to characterize the extracted polymer. The efficient marine bacterial isolates were identified by the phylogenetic analysis of the 16S rRNA genes as *Tamlana crocina*, *Bacillus aquimaris*, *Erythrobacter aquimaris*, and *Halomonas halophila*. The optimization of PHB accumulation by *E. aquimaris* was achieved at 120 h, pH 8.0, 35 °C, and 2% NaCl, using glucose and peptone as the best carbon and nitrogen sources at a C:N ratio of 9.2:1. The characterization of the extracted biopolymer by Fourier-transform infrared spectroscopy (FTIR), Nuclear magnetic resonance (NMR), and Gas chromatography-mass spectrometry (GC-MS) proves the presence of hydroxyl, methyl, methylene, methine, and ester carbonyl groups, as well as derivative products of butanoic acid, that confirmed the structure of the polymer as PHB. This is the first report on *E. aquimaris* as a PHB producer, which promoted the hypothesis that marine rhizospheric bacteria were a new area of research for the production of biopolymers of commercial value.

## 1. Introduction

Petroleum-based plastics have serious ecological and social impacts because of their non-degradable nature and the leaching of carcinogenic substances when exposed to scratch or heat [1]. The bio-based materials provide copious chances to design composites with advanced bio-functionalities and minimize our reliance on petroleum-based resources, such as combining spider silk proteins with the cellulose nanofibrils [2], flow-assisted organization of cellulose nanofibrils into macroscale fibers to exceed their mechanical properties [3,4], and the synthesis of multifunctional hollow porous carbon capsules that are encapsulated with silver nanoparticles for antimicrobial and anti-bioadhesion applications [5]. Lately, humanity has realized the necessity of supplanting non-biodegradable ordinary plastics with eco-friendly bioplastic options [6,7]. Polyhydroxyalkanoates (PHAs) are intracellular biopolymers that take on an extraordinary significance because of their physicochemical, biodegradable, and biocompatible properties [8]. PHAs are accumulated in bacteria, archaea, yeasts, and fungi under stress conditions, where the carbon source is plentiful and other nutrients are limited, to be the key mechanism for microbial survival [9]. Despite there being around 150 diverse PHA structures recognized so far, poly-β-hydroxybutyrate (PHB) has attracted more attention than the other types because of its physical, mechanical, and immunologically properties, which make it a strong candidate for use in various applications in agriculture, food, and medical fields [10,11]. Advanced uses of bioplastics are still evolving, especially the development of food-borne pathogen-resistant bioplastics and the production of bioplastic nanoparticles used in the development of drug delivery systems [12,13]. PHB is synthesized through the condensation of two Acetyl-CoA molecules to Acetoacetyl-CoA by the Acetyl-CoA-acetyltransferase, which is then reduced to (*R*)-3-hydroxybutyrate-CoA by the acetoacetyl-CoA-reductase, and then the polymerization of (*R*)-3-hydroxybutyrate-CoA to PHB by the PHB synthase [14]. Microbial PHB production and its chemical characterization have not been investigated in detail [15,16]. The industrial bioprocess of PHB is still developing, and microbial resources are still being explored for extensive production and commercialize issues [8]. The exploitation of renewable materials as a source of valuable commodities as microbial natural products have acquired great attention recently, as this offers an alternative to the environmental problems caused by this waste. Therefore, the concept of utilizing date palm waste to develop low-cost PHB fermentation has been receiving extensive attention [17]. As microbial strains accumulate PHB as a survival mechanism, an ecosystem with extreme stress conditions, such as a marine environment could be containing superior PHB producers [18]. Marine bacteria have recently attracted attention for this purpose, including *Alteromonas lipolytica*, *Bacillus megaterium*, *Halomonas campaniensis*, *Pseudomonas* spp., *Aeromonas* spp., *Cupriavidus* spp. *Archaebacteria*, *Methylobacteria*, and *Pseudomonas* [11]. The Red Sea environment has not been explored enough for the purposes of its microbial diversity, biotechnological processes, and biopolymer production [19]. Compared to other marine environments, the Red Sea is considered an extraordinary marine ecosystem because of its physical and geochemical parameters and its relatively secluded location away from other marine environments [20]. The rhizosphere marine bacteria isolated from these conditions are a major source of novel and unique metabolites [21]. Mangroves a unique wetland ecosystem in Saudi Arabia that play various ecological roles, but that are currently not effectively managed for research and development. They have a huge root system with a high diversity of microbial communities able to produce unique bio-products [6]. The marine bacteria, *Vibrio* spp., *Bacillus thuringiensis* and *Bacillus* sp. capable of effectively producing PHB, were isolated from mangrove soil [9,21,22]. The polymer chain of PHB is formed by ester bonds forming between the carboxyl group and an available hydroxyl group of nearby monomers [23]. To determine their functional groups and chemical structure, the biopolymers were characterized by various instrumental techniques, Fourier-transform infrared spectroscopy (FTIR), Nuclear magnetic resonance (NMR), and Gas chromatography-mass spectrometry (GC-MS) [14,24]. Accordingly, this study focuses on the isolation and screening of PHB-producing bacteria from the mangrove s’ rhizospheres in the Red Sea, Saudi Arabia, and explores the means of optimizing their biopolymer production, as well as studying its chemical characterization.

## 2. Results and Discussion

### 2.1. Isolation of Marine Bacteria

A total of 48 bacterial isolates were obtained from the mangrove rhizospheres of the two Red Sea coasts; Al-Madhaya and Al-Marabi. These sites were selected because they had not been explored previously. The highest number of bacteria were isolated from the mangrove rhizosphere from Al-Marabi coast (31 isolates), and the lowest number (17 isolates) from Al-Madhaya coast. The appearance of isolates in the various habitats may be affected by the predominant environment and culture conditions [6,25]. Mangrove grows in the southern region of Saudi Arabia is generally dense because of the tropical climate, higher rainfall, and less saline seawater [26]. However, sewage disposal on the Al-Madhaya coast may be the reason for negative effects on the mangroves and microbial communities in their rhizosphere. All of the bacterial isolates were assigned code numbers based on their site of isolation.

### 2.2. Qualitative Screening and Selection of Poly-β-hydroxybutyrate-Producing Marine Bacteria

**Figure 1:**
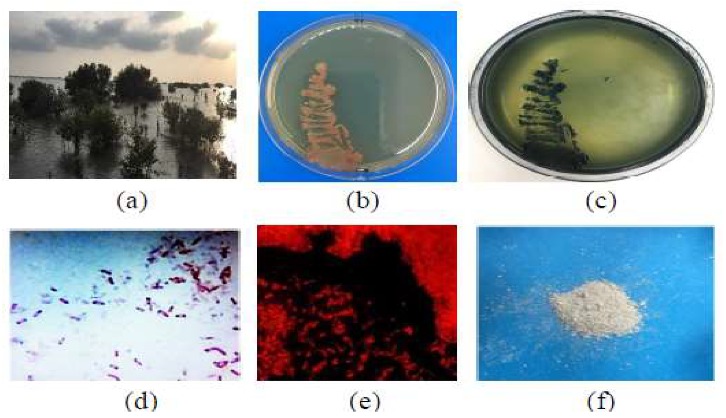
Screening of promising marine bacterial isolate, KKU-MD13, for poly-β-hydroxybutyrate (PHB) production. (**a**) Mangaroves in Al-Madhaya coast; (**b**) bacterial culture; (**c**) plate staining with Sudan Black-B; (**d**) slide staining with Sudan Black-B; (**e**) slide staining with Acridine orange; (**f**) powder of extracted PHB.

The bacterial isolates were primarily screened using Sudan Black-B stain. The colony staining method was employed for the rapid detection of PHB, followed by microscopic examination. Out of the 48 bacterial isolates, only 14 were able to accumulate the PHB granules. The second screening was conducted by Acridine orange dye under a fluorescent microscope (Figure 1). The results show that four bacterial isolates have a high ability to produce PHB; KKU-MD12 and KKU-MD13 from Al-Madhaya coast, and KKU-MR35 and KKU-MR42 from the Al-Marabi coast. These findings were considered additional evidence for the specificity of the Sudan Black-B and Acridine orange staining approach to screen new bacterial isolates producing an intracellular lipid biopolymer. The dark blue colored colonies stained by Sudan Black-B [27], and yellow colored granules inside the bacterial cells by Acridine orange [28] were observed in similar studies, which was taken as a positive indication for PHB production. The lipophilic staining with Sudan Black-B has a high sensitivity in PHB screening, it reveals clearly the intracellular lipid matter in the form of cytoplasmic inclusions or materials associated with the structural elements of the cells, which previously have not been seen or even suspected [29]. On the other hand, using the fluorescent dye Acridine orange, differentiation between the living cells, fat droplets, and dead cells becomes very easy, because the fluorescence of the different wavelengths appears after staining [30]. Our investigation with respect to PHB-producing bacteria demonstrates that the bacterial communities isolated from the samples of mangrove rhizospheres were not quite the same as those recorded in other marine sources. On account of the high rate of evaporation, absence of river inflows, low degree of sedimentation, and low nutrients, the Red Sea was considered to be an exceptional environment, which encourages the biodiversity of microbial species to be able to accumulate PHB in order to survive under starvation conditions [31].

### 2.3. Bacterial Identification Using 16S rRNA Gene and Phylogenetic Analysis

**Figure 2:**
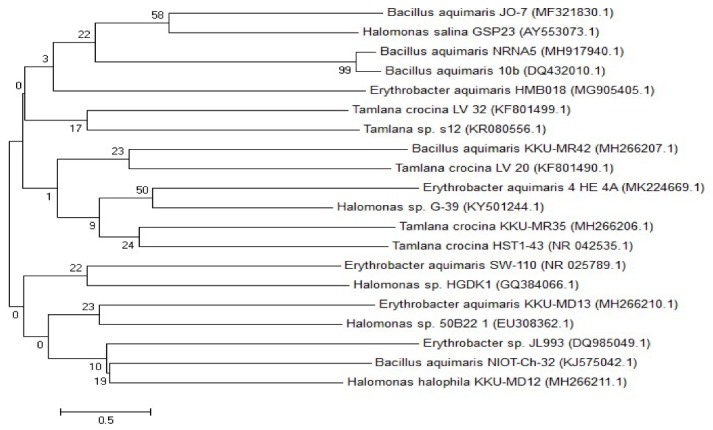
Phylogenetic tree based on 16S rRNA nucleotide sequences of the bacterial isolates *Tamlana crocina*, *Bacillus aquimaris*, *Erythrobacter aquimaris*, and *Halomonas halophila*, with other sequences of published strains generated by the neighbor-joining method using MEGA 5.0 software. The scale bar corresponds to a 0.05 nucleotide substitution per sequence position. The numbers at the nodes indicate the levels of bootstrap support (%) based on 1000 resampled data sets. The number in parentheses represents the accession number in GenBank.

The molecular identification of bacteria by the amplification of the 16S RNA gene and other genotypic approaches are accurate modern tools compared to the traditional identification methods using staining and biochemical tests [27,28]. The potent four PHB-producing isolates were submitted for DNA extraction and PCR amplification of their 16S rRNA gene. The obtained sequences were deposited in the GenBank under specific accession numbers (Table 1), and the relationship between them was determined in the phylogenetic tree (Figure 2). The main purpose of integrating all of the sequences into a single tree was to investigate the molecular similarity of the bacterial strains that share the same environment, but can exhibit different physiological behaviors. The data reveal that all of the isolates originated from the same ancestor and share a high genetic similarity, along with ribosomal genes, which might reflect the genetic similarity in the whole genome, indicating that these isolates were a native of mangrove rhizospheres. Of the four PHB-producing bacterial isolates identified, two isolates belonged to the Proteobacteria phylum, *Erythrobacter aquimaris* and *Halomonas halophile*, one isolate, *Tamlana crocina*, belongs to the phylum Bacteroidetes, and *Bacillus aquimaris* is related to phylum Firmicutes. These results were in harmony with the finding of Quillaguaman et al. [32], who mentioned that marine ecosystems were highly variable; consequently, their diverse microbes have gained more consideration as a possible producer of biopolymers. They also found that the most halophilic bacteria able to accumulate PHB belonged to the Proteobacteria phylum, which was predominant in marine environments. The results of the current investigation revealed that the bacterial strains isolated from the mangrove environment of the Red Sea were a potent source of PHB. About 50 PHB-producing bacteria were isolated from the mangrove soil [21]. Also, the microbes in the marine environment remain undiscovered, despite the fact that they could possibly synthesize significant quantities of PHB because of their unique genes and enzymes [6].

### 2.4. Quantitative Screening of PHB-Producing Marine Bacteria

**Figure 3:**
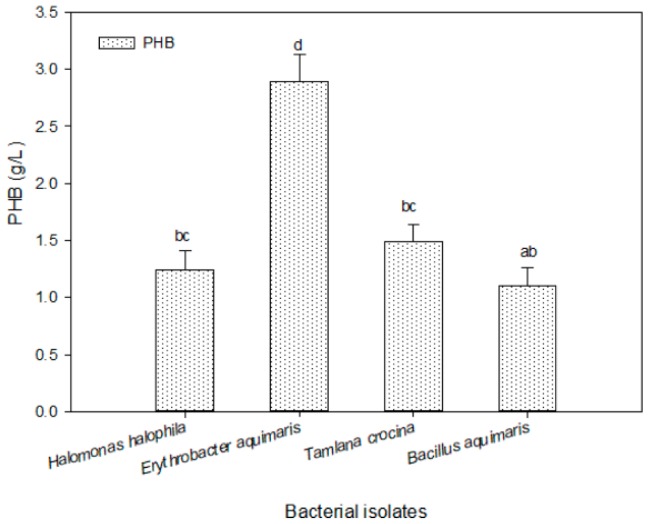
Quantitative screening of promising marine bacterial isolates; *Halomonas halophila* (KKU-MD12), *Erythrobacter aquimaris* (KKU-MD13), *Tamlana crocina* (KKU-MR35), and *Bacillus aquimaris* (KKU-MR42) for PHB production. The values set by oneself letter(s) on the same column were not significantly different.

Depending on the high PHB production by qualitative screening using Sudan Black B stain and acridine orange for 48 isolates, four promising isolates were subjected to quantitative screening through submerged fermentation. As it is clear in Figure 3, the highest production of PHB was recorded by the bacterial isolates *Erythrobacter aquimaris* (2.90 g/L), obtained from Al-Madhaya coast, followed by *Tamlana crocina*, isolated from Al-Marabi coast (1.49 g/L). PHB-producing bacteria *Vibrio* spp., *Bacillus thuringiensis*, *Bacillus* sp., and *Vibrio* MK4 were isolated from mangrove s’ rhizospheres [9,21,22,33]. 

### 2.5. Optimization of PHB Production by E. Aquimaris

The optimization of PHB production by *E. aquimaris* was conducted by an approach of one factor at a time. The outcomes of the improvement parameters revealed that the optimization process plays an important role in the production rate of PHB.

#### 2.5.1. Effect of Fermentation Period on PHB Production

**Figure 4:**
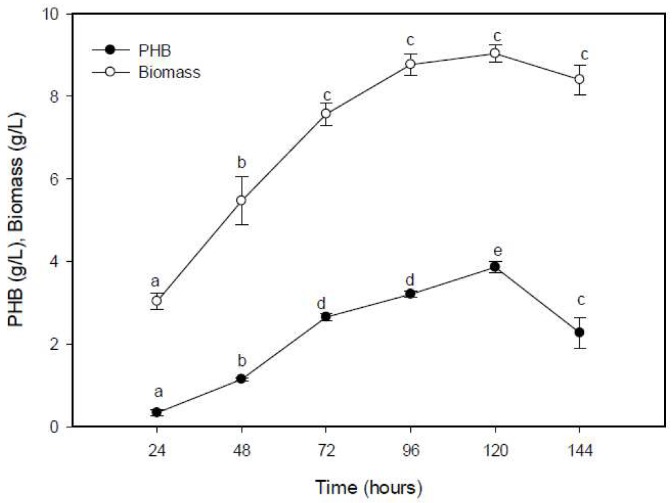
Effect of fermentation period on the PHB production by *E. aquimaris*. The values set by oneself letter(s) on the same line were not significantly different.

The PHB production was increased gradually from 8.6% in the initial 24 h of incubation to 73% at 72 h at the beginning of the logarithmic phase (Figure 4). A significant result of 3.86 g/L was obtained after 120 h at the stationary phase of growth. There was a correlation between the bacterial growth and the accumulation of PHB, as recorded by Gomaa [34], who noted that 96 h of cultivation was an optimum period for the maximum PHB production by *Bacillus subtilis* and *E. coli.* PHB accumulation by a specific bacterial species has a direct relationship with its biomass, which inevitably reduced as the biomass was reduced as the carbon was depleted, leading to the consumption of PHB [35]. The various cultivation periods have been recorded in the literature as being optimal for the bacterial accumulation of PHB; for example, 48 h of incubation for *Rhizobium alti*, *Pseudomonas stutzeri*, and *Bacillus* sp. [35,36]; 40 h of incubation for the sponge isolate, *Bacillus subtilis* [37]; and 24 h by halophilic *Bacillus megaterium* [38]. On the other hand, as confirmed previously, the PHB production was diminished after the ideal fermentation period [25,39,40]. They reported that the marine bacteria *Bacillus* sp. CS-605, *Bacillus megaterium*, and *Vibrio harveyi* accumulated PHB as a food reserve, which was degraded by depolymerase when external carbon sources were exhausted at the stationary phase, thereby promoting its survival.

#### 2.5.2. Effect of Initial pH on PHB Production

**Figure 5:**
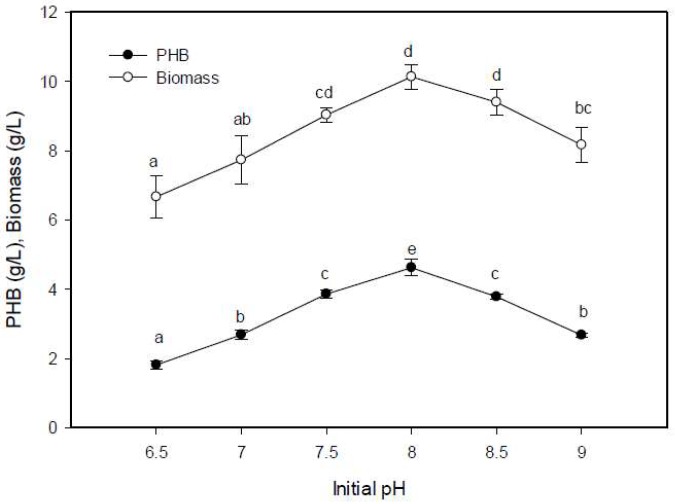
Effect of initial pH on the PHB production by *E. aquimaris*. The values set by oneself letter(s) on the same line were not significantly different.

The optimum accumulation of the PHB and bacterial biomass were obtained at pH = 8.0, and reached 4.62 g/L (Figure 5), which was in agreement with the findings of Kalaivani and Sukumaran [28]. They recorded the optimal production of PHB by *Saccharococcus thermophilus* to be at pH = 8, producing 13.2 g/L. Additionally, PHB production by *Vibrio harveyi* 284 to be 1.2 g /L at pH = 8.0 [40]. The production of PHB declined by 18% in the alkaline condition of pH = 9, while in the acidic condition of pH = 6.5, there was a 60.8% reduction in PHB production. PHB accumulation was supported at pH = 7–9, but was dramatically reduced outside this range [18]. The pH exhibited a potent influence on the PHB accumulation because of its effect on the bioavailability of the trace elements [34] and the regulatory enzymes responsible for the synthesis of PHB, β-ketothiolase, acetoacetyl-CoA reductase, and PHA polymerase [25].

#### 2.5.3. Effect of Incubation Temperature on PHB Production

**Figure 6:**
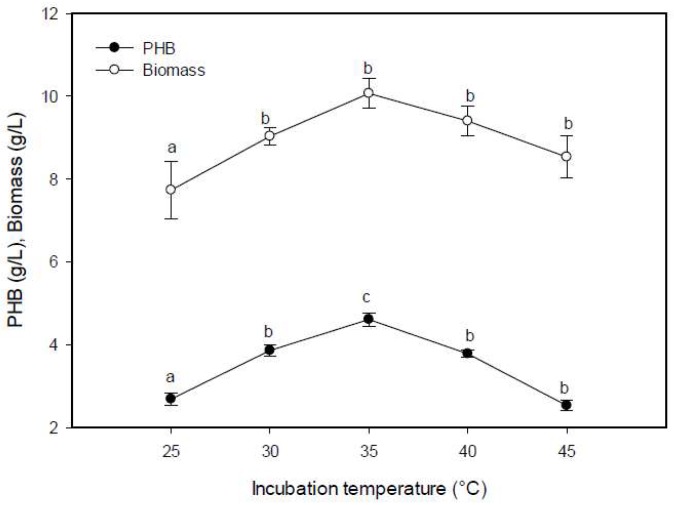
Effect of incubation temperature on the PHB production by *E. aquimaris*. The values set by oneself letter(s) on the same line were not significantly different.

The results indicate a considerable relationship between PHB production and incubation temperature. The maximum PHB production and bacterial biomass occurred at 35 °C (Figure 6), which in accordance with other research [34,36]. The effect of temperature on PHB production was varied among the genera. A temperature of 30 °C was optimal for the highest production of PHB by *Nacardiopsis potens*, *Rhizobium elti*, *Pseudomonas stutzeri*, and *Vibrio harveyi* [27,35,40], and 50 °C was the best for marine *Saccharococcus thermophilus* [28]. The effect of the temperature on the variation in PHB production can be due to the fact that temperatures other than the optimal one reduced the activity of the enzymes responsible for PHB synthesis [41].

#### 2.5.4. Effect of NaCl Concentration on PHB Production

**Figure 7:**
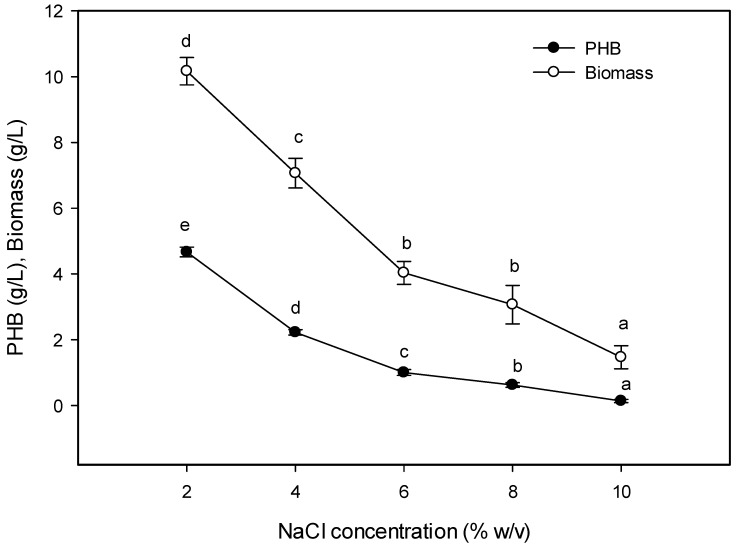
Effect of NaCl on the PHB production by *E. aquimaris*. The values set by oneself letter(s) on the same line were not significantly different.

The maximum PHB production was recorded at 2% (*w*/*v*) NaCl, reaching 4.67 g/L (Figure 7). This result was partially proportioned with the recorded salinity of the Al-Madhaya coast samples (35.1 ppt), which indicated that this isolate belongs to slight halophiles. Most of the marine microbes were slight and moderate halophiles, while extreme halophiles inhabit hypersaline environments such as the deep-sea and underground salt mines [42]. This finding reflects the necessity of controlling the salinity of the medium within a proper range to prevent high osmotic stress exerting an effect on PHB production [39], where increasing the salt to 6% was caused by a decline in PHB production by 78.58%. The salt concentration affecting the PHB accumulation was varied among the bacterial strains: 5% for *Vibrio proteolyticus* [7], 2.5% for *Nacardiopsis potens* [27], 3% for *Bacillus* sp. *CS-605* [25], 2% for *Vibrio harveyi* [40], and 1.5% for *Vibrio azureus* [18].

#### 2.5.5. Effect of Carbon Sources on PHB Production

**Figure 8:**
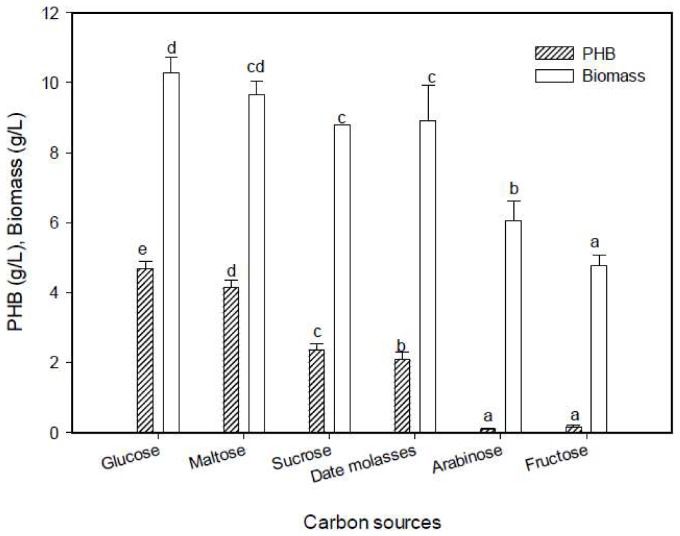
Effect of carbon source on the PHB production by *E. aquimaris*. The values set by oneself letter(s) on the same column were not significantly different.

The carbon source is a major nutritional factor playing an essential role in PHB production, as the bacteria stores it in the form of PHB granules. The results show that glucose was the preferred carbon source for PHB production (Figure 8). It was exhibited as a carbon source that promotes the bacterial growth and production of PHB by *Vibrio azureus* BTKB33, *Bacillus megaterium*, *Bacillus* sp., and *Alcaligens eutrophus* [18,38,41,43]. In spite of maltose being the second-most useful carbon source for *E. aquimaris* to synthesize PHB, only a 11.53% reduction in production was observed, it was the most suitable carbon source for the accumulation of PHB for *Bacillus* sp. CS-605 [25]. Few studies have focused on the production of PHB from date palm molasses, a cheap by-product in Saudi Arabia [17]. Date molasses is an important source of sugar, mainly glucose, fructose, and sucrose [44]. From our results, date molasses contained 82.3% total sugars, with 44% glucose, 37% fructose, and 9.2% sucrose. An attempt to use it as a cheap carbon source for PHB production yielded only 44.87% PHB compared with glucose. The ability of the bacterium to utilize different complex carbon sources was dependent on several factors, such as the nature of the substrate and the type of enzymes synthesized [39]. However, cane molasses as a suitable substrate for PHB by *Bacillus subtilis* was used [34], and the high accumulation of PHB by *Bacillus megaterium* and *Lactococcus lactis* was achieved by using a by-product of biodiesel as the substrate [45]. The other carbon sources tested did not facilitate either PHB production or bacterial growth. The PHB production was minimal by arabinose and fructose, where the production was reduced to 2.35% and 3.63%, respectively, as mentioned previously [33].

#### 2.5.6. Effect of Nitrogen Sources on PHB Production

**Figure 9:**
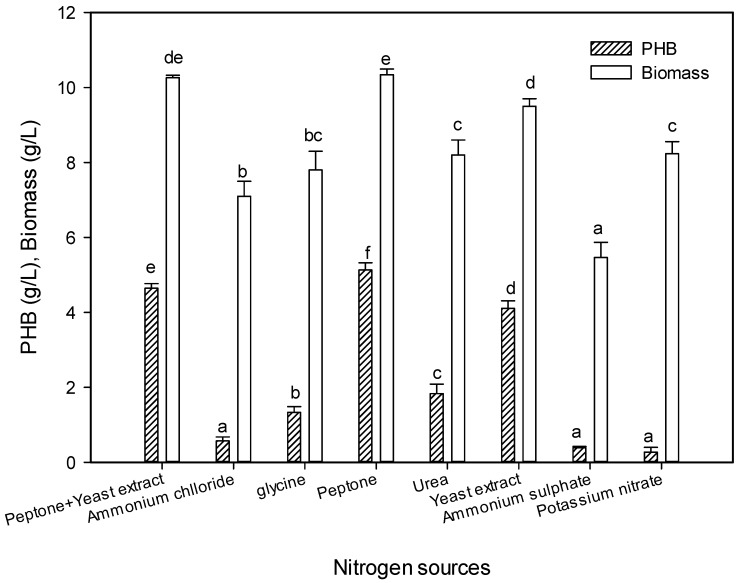
Effect of nitrogen source on the PHB production by *E. aquimaris*. The values set by oneself letter(s) on the same column were not significantly different.

The nitrogen source is the most limiting factor for PHB production, where bacterial strains need a high concentration of nitrogen at the start of the fermentation process in order to produce high biomass, and after that start creating the polymer when nitrogen depletes as a survival mechanism [46]. The effect of different organic and inorganic nitrogen sources on PHB production was studied (Figure 9). The nitrogen source in the production medium (peptone + yeast extract) was replaced with tested nitrogen sources at the same concentration. The substantial PHB production using peptone may be attributed to the presence of complex organic nitrogen sources, as mentioned by Patel et al. [47], who found that PHB production took place when the nitrogen was limited and complex. In contrast, peptone was found to be the least supportive of PHB production in *Rhizobium elti* E1 and *Pseudomonas stutzeri* E114, and the ammonium sulfate was optimal, which proves the possibility of more pathway to synthesize PHB [35]. Moreover, the low nitrogen content of peptone brought an increase in the C:N ratio, which supported a higher PHB production [41]. It appears that the control medium (yeast extract and peptone) was not suitable for PHB production, as mentioned previously [8,28]. The yeast extract was the next favorable nitrogen source, while other nitrogen sources significantly reduced PHB production. There were significant differences between PHB production using organic and inorganic nitrogen sources. The highest reduction in PHB production was recorded using inorganic sources, which reached only 11.11%, 7.52%, and 5.32% for ammonium chloride, ammonium sulfate, and potassium nitrate, respectively, corresponding with Hungund et al. [46].

#### 2.5.7. Effect of C:N Ratio on the PHB Production

**Figure 10:**
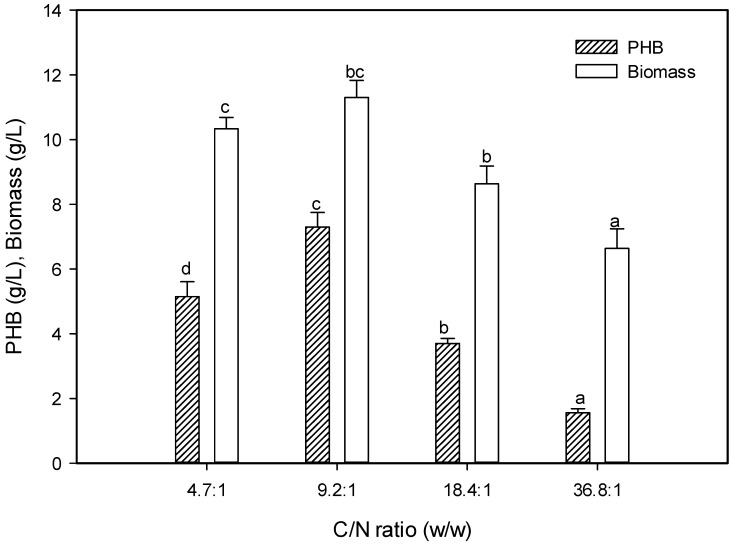
Effect of C:N ratio on the PHB production by *E. aquimaris*. The values set by oneself letter(s) on the same column were not significantly different.

To investigate the effect of the different C:N (*w*/*w*) ratios on PHB production, a constant initial peptone concentration with different initial glucose concentrations was prepared at 4.7:1, 9.2:1, 18.4:1, and 36.8:1. The production of PHB was increased when the C:N ratio in the medium was 9.2:1 and reached 7.3 g/L (Figure 10). During the standard conditions for microbial fermentation, the cells synthesize protein, while, under uneven nutrients conditions, a few microbes can accumulate PHB granules to survive [8]. The PHB production and bacterial growth decreased significantly at a high C:N ratio. This finding reveals the role of the nitrogen limitation, which is inversely proportional to the production of PHB [47]. Despite the universality of a low-nitrogen medium being preferential for PHB production, the C:N ratio was diverse among the bacterial strains. A C:N ratio of 20:1 was found to support bacterial PHB production [36], while the C:N ratio at 2:0.2 was reasonable for PHB accumulation by marine bacteria, *Nacardiopsis potens* [27]. Moreover, at a high C:N ratio, the PHB accumulation, and bacterial growth were decreased as a result of substrate inhibition, where the glucose has an inhibitory effect at a high level, which influences the specific growth rate and PHB production [47].

### 2.6. Chemical Characterization of Extracted PHB

#### 2.6.1. Fourier-Transform Infrared Spectroscopy Analysis

**Figure 11:**
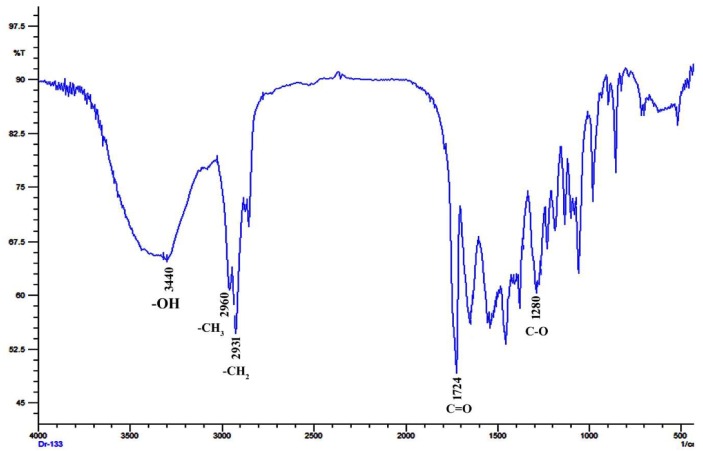
Fourier-transform infrared spectrum of PHB extracted from *E. aquimaris*. The absorption bands at 3440, 2960, 2932, 1724, and 1280 cm^−1^ corresponding to OH, CH_3_, CH_2_, C=O, and C–O groups.

An FTIR analysis was carried out to identify the functional groups present in the PHB extracted from *E. aquimaris* (Figure 11). The carbonyl group (C=O) is a common feature in all of the structures of PHAs [41]. The FTIR spectrum of the extracted PHB shows a strong band at 1724 cm^−1^ for the carbonyl (C=O) stretching of the ester group, which corresponds to peaks recorded for the standard PHB. A significant peak of the C=O group at 1728 cm^−1^ for the PHB of *Bacillus* sp. CS-605 was recorded [25], while a band at 1736 cm^−1^ was recorded for the PHB from *Vibrio azureus* BTKB33, indicating the presence of C=O group [18]. Also, the FTIR spectra of the PHB extracted from halotolerant mangrove isolate, *Bacillus* MG12, showed a peak at 1736 cm^−1^, corresponding to a C=O stretch of the ester group [22]. Furthermore, the other functional groups detected by the FTIR spectrum were consistent with the results presented in the literature. The peaks at 3440 cm^−1^ indicated a strong stretching H bond created by the terminal OH groups [48]. The peaks at 2931 and 2960 cm^−1^ were assigned to C–H stretching methyl and methylene groups, respectively; these were well comparable with the results of Sathiyanarayanan et al. [37]. The FTIR spectrum of the extracted PHB also showed a band at wave number 1280 cm^−1^, corresponding to the C–O group, which is in accordance with Likitha et al. [49].

#### 2.6.2. Nuclear Magnetic Resonance Spectroscopy Analysis

**Figure 12:**
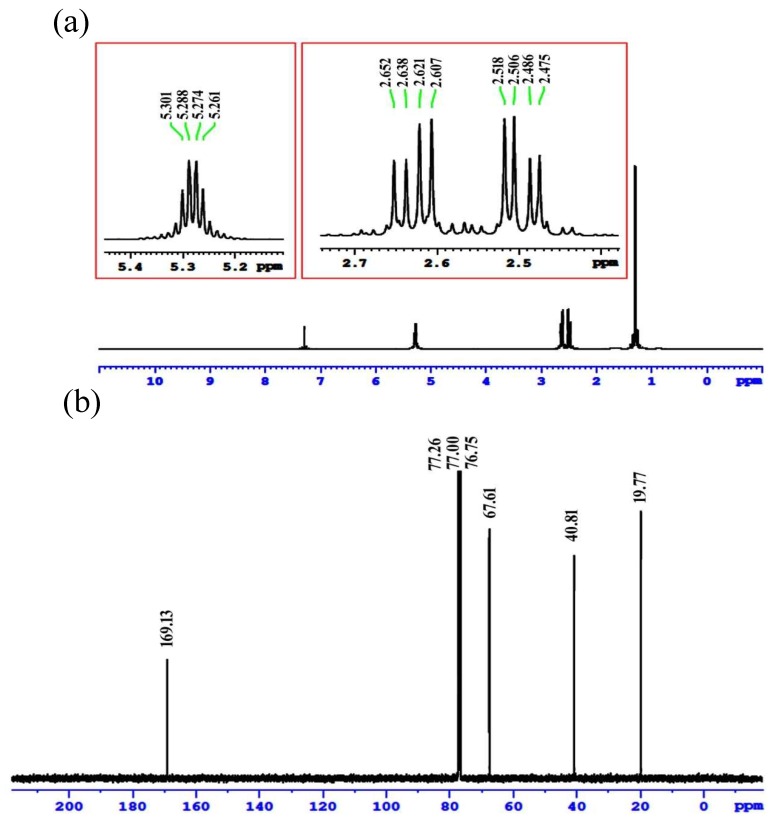
Nuclear Magnetic Resonance (NMR) analysis of PHB extracted from *E. aquimaris*. (**a**) ^1^H NMR spectrum of PHB shows signals at chemical shift *δ* 1.31 (d, CH_3_,), 2.472 (q, CH_2_,), and 5.26–5.30 ppm (m, CH,). (**b**) ^13^C NMR spectrum of PHB shows signals at 169.13, 67.61, 40.81, and 19.77 ppm for carbon atom of CO, CH, CH_2_, and CH_3_ groups.

NMR spectroscopy is a significant strategy for checking if the polymer structure effectively describes the structure of PHB [50]. The ^1^H NMR spectrum of the extracted PHB exhibited signals of chemical shift at *δ* = 1.31 ppm as a doublet for methyl group, and a pair of quadruplets at *δ* = 2.47–2.65 ppm, which is characteristic of a methylene group (CH_2_) linked to the carbonyl group (Figure 12a). Multiple signals appear at *δ* = 5.26–5.30 ppm, characteristic of a methine group (–CH). These results can be summarized as follows ^1^H NMR (500 MHz, CDCl_3_): δ 1.31 ppm (d, CH_3_, *J* = 6.4 Hz), 2.47–2.65 ppm (DQ, CH_2_, *J* = 7.6 and 5.6 Hz), and 5.26–5.30 ppm (m, CH, *J* = 5.6 Hz). The ^1^H NMR spectrum of the PHB of *Vibrio harveyi* exhibited three signals for different groups, namely methyl, methylene, and methane, at 1.21, 2.56, and 5.22 ppm, respectively [40]. Moreover, the ^1^H NMR resonances of the polymer of marine *Bacillus megaterium* appeared at 1.6 ppm for methyl, 2.4 ppm for methylene, and 5.2 ppm for the methine group, confirming its structure as a PHB [38]. Additionally, the NMR spectra of the PHB from marine *Bacillus cereus* and *Bacillus subtilis* was extrapolated with major peaks at 1.23, 2.5, and 5.2 ppm, as the resonance absorption of the methyl, methylene, and methine groups, respectively [24,37].

The ^13^C NMR spectrum displayed signals at (1) 169.13 ppm for the quaternary carbon of the carbonyl group; (2) 67.61 ppm for the CH; (3) 40.81 ppm for the CH_2_ group, which appeared close to the carboxyl group; and (4) 19.77 ppm could be attributed to the methylenic carbon (Figure 12b). The same results were observed for the ^13^C NMR spectrum of the PHB extracted from *Vibrio harveyi*, which showed chemical shift signals at 169.14, 67.61, 40.79, and 19.76 ppm, corresponding to the carbonyl, methine, methylene, and methyl groups, respectively [40]. Moreover, the absorbance of the polymer extracted from *Lysinibacillus sphaericus* was 169.143, 67.656, 40.864, and 19.787 ppm for the carbonyl, methine, methylene, and methyl resonance, respectively, which confirmed the presence of PHB [50].

#### 2.6.3. Gas Chromatography-Mass Spectrometry Analysis

**Figure 13:**
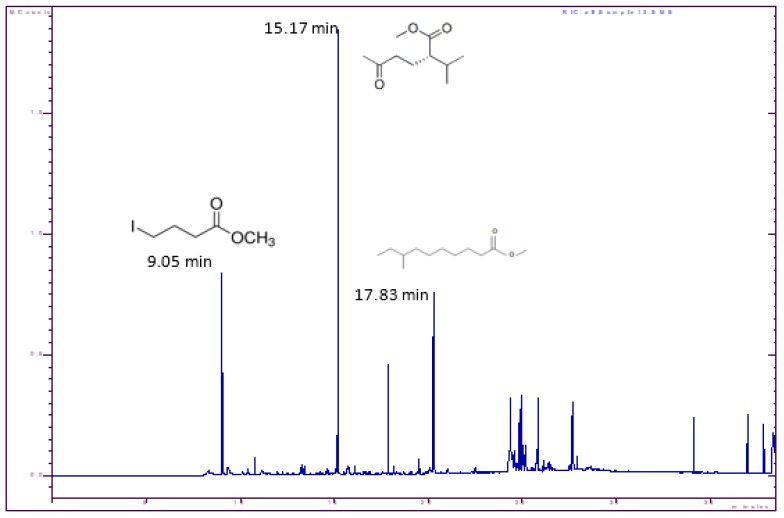
Gas chromatography–mass spectrometry chromatogram of PHB extracted from *E. aquimaris* shows three monomeric compositions of PHB; Butanoic acid, 4-Iodo-, methyl ester; Hexanoic acid, 2-(1-methylethyl)-5-oxo-, methyl ester; and Decanoic acid, 8-methyl-, methyl ester.

**Figure 14:**
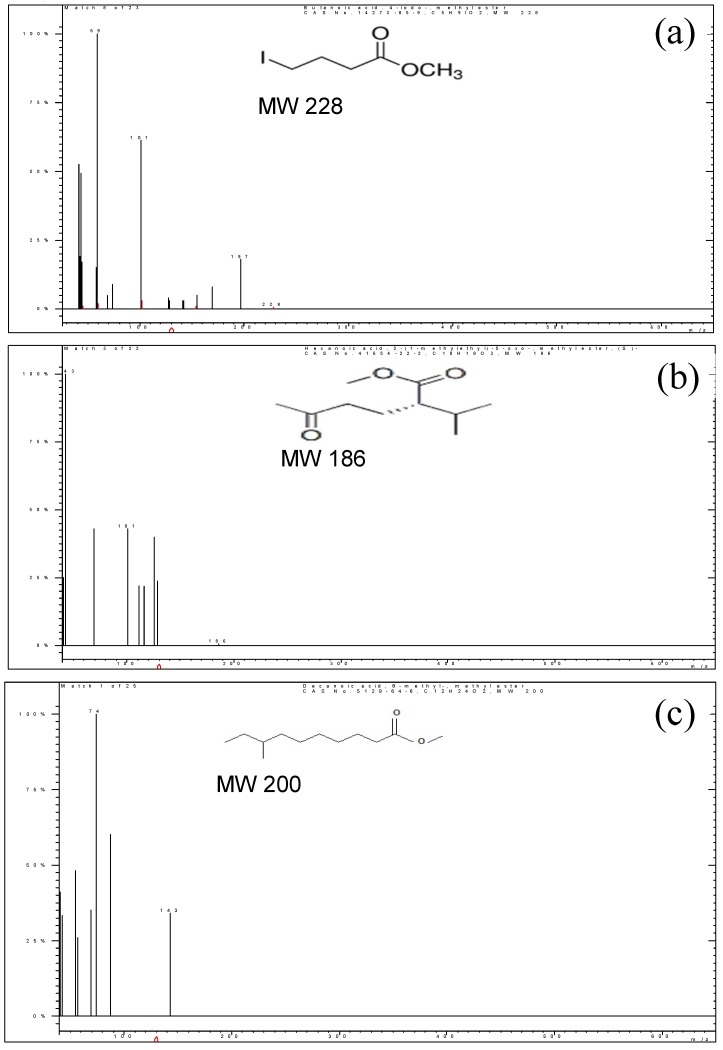
Mass spectra of (**a**) Butanoic acid, 4-Iodo-, methyl ester; (**b**) Hexanoic acid, 2-(1-methylethyl)-5-oxo-, methyl ester; (**c**): Decanoic acid, 8-methyl-, methyl ester.

The PHB polymer from *E. aquimaris* was also analyzed by GC-MS to determine the monomeric composition of PHB by its conversion to volatile hydroxycarboxylic acid methyl esters by acidic methanolysis [14]. Three prominent peaks with Rt values of 09.05, 15.17, and 17.83 min corresponding to three different derivative products of butenoic acid were detected (Figure 13 and Figure 14)—butanoic acid, 4-iodo-, methyl ester, hexanoic acid, 2-(1-methylethyl)-5-oxo-, methyl ester, decanoic acid, 8-methyl-, and methyl ester, confirming the presence of PHB. These results were compatible with the GC-MS analysis of the PHB extracted from *Bacillus subtilis*, which revealed the major peak resembling methyl 3-hydroxybutyrate, which confirms the PHB structure [37]. The GC-MS spectrum of the methyl ester derivative of the extract of PHB obtained from *Bacillus megaterium* exhibited a signal at 23.3 min, corresponding to the methyl ester methyl ether 3-hydroxybutyric acid [45]. Also, the PHB extracted from *Lysinibacillus sphaericus* contains a 3-hydroxy functional group and the presence of methyl esters of hydroxyl butyrate [50].

## 3. Materials and Methods

### 3.1. Sample Collection

The marine samples of the mangrove rhizospheres were collected from the Red Sea, southwestern coast of Saudi Arabia, from two sites, Al-Marabi coast (42°43′44.1″ E, 16°39′59.5″ N) and the Al-Madhaya coast (42°39′59.3″ E, 16°47′22.3″ N). Three replicates of each mangrove rhizosphere were taken from each site (at a depth of 10 cm), in a sterilized glass screw cap bottle, and then kept at 4 °C until processed. The temperature, pH, and salinity of the samples were measured in real-time during sampling using Portable Meters (OAKTON Instruments, Vernon Hills, IL, USA). The pH was 7.9–8.1 for all of the samples, while the temperature was about 33–35 °C. The salinity of the Al-Marabi coast samples were 36.3 ppt, whereas the Al-Madhaya samples were 35.1 ppt. The date molasses of Khalas dates were obtained from the Nadec Company, Riyadh, Saudi Arabia. The sugars content was analyzed using high-performance liquid chromatography (Agilent Technologies, 1200 Model Infinity, Burnsville, MN, USA) [51]. 

### 3.2. Isolation of PHB-Producing Marine Bacteria

The samples were sequentially diluted and 100 µl of the dilution was spread on the Zobell marine agar [52]. The medium contained (g/L) the following: peptone 5.0, yeast extract 1.0, ferric citrate 0.1, NaCl 19.45, MgCl_2_ 8.8, Na_2_SO_4_ 3.240, CaCl_2_ 1.8, KCl 0.55, NaHCO_3_ 0.160, KBr 0.080, SrCl_2_ 0.034, H_3_BO_3_ 0.022, Na_2_SiO_3_ 0.004, NaF 0.0024, NH_4_NO_3_ 0.0016, Na_2_HPO_4_ 0.008, and agar 15.0 (pH = 7.5), and was enhanced with 1% glucose as the carbon source. All of the inoculated plates were incubated at 35 °C for 72 h at 150 rpm in a shaking incubator (Shell Lab, SSI5, Cornelius, NC, USA). The bacterial colonies that appeared were purified by using a repetitive streaking method, and were preserved at 4 °C on Zobell marine agar slants.

### 3.3. Screening of PHB-Producing Bacteria

#### 3.3.1. Screening by Sudan Black-B Stain

To evaluate the capacity of the marine isolates to accumulating PHB, the primary screening by Sudan Black B staining was conducted. The culture plates were covered with an alcoholic solution of Sudan Black B (0.05%) for 30 min, and then washed with ethanol. The colonies that appeared in black or dark blue were considered to be PHB producers. A smear of each isolate was also stained with Sudan Black B (0.3%, *w*/*v*) for 20 min, then immersed in xylene before being stained with aqueous safranin (0.5%, *w*/*v*). The slides were then washed with distilled water and air-dried [27].

#### 3.3.2. Screening by Fluorescence Staining Method

The bacterial isolates showing a positive result in the previous test were screened for a second time with a fluorescent dye, Acridine orange. First, 50 µL of dye was added to 10 µL of 72 h old bacterial culture, and incubated for 30 min at 35 °C. The cultures were centrifuged for 5 min at 6000 rpm, after that the smear was prepared and observed by a fluorescent microscope (Nikon, Eclipse, e400, Tokyo, Japan). The appearance of yellow-colored granules inside the cell was taken as a positive indication of PHB accumulation [16].

#### 3.3.3. Quantitative Screening of PHB Production

The promising isolates were quantitatively screened for the accumulation of PHB. First, 1 mL of a 24 h old culture (2 × 10^8^ CFU/mL) was inoculated in a 250 mL Erlenmeyer flask containing 50 mL of the Zobell marine broth, and incubated for 72 h at 35 °C/150 rpm, then the extracted PHB was estimated.

### 3.4. Extraction and Quantitative Estimation of PHB

To extract the PHB granules, the dry bacterial pellets were washed with acetone and ethanol, then suspended in 10 mL of 4% NaClO and incubated for 60 min at 37 °C. The mixture was centrifuged (5000 rpm for 30 min) to sediment the PHB granules. The polymer was washed with acetone and ethanol followed by dissolving in hot chloroform. The cell residues filtered out using Whatman filter paper so that only PHB was presented in chloroform solution, which evaporated in a hot air oven at 40 °C, leaving the PHB powder [53]. To quantify the PHB, the stock solution of the extracted PHB was prepared by dissolving 50 mg PHB in 10 mL of concentrated sulfuric acid. The standard solutions of pure PHB were set up by diluting aliquots of the stock solution (20 to 200 µg/ mL). Then, 10 mL of sulfuric acid was added to the extracted PHB in a water bath (100 °C/10 min) to convert it into crotonic acid. The absorbance of the extracted PHB has recorded a spectrophotometry (PerkinElmer Lambda 25 UV/VIS spectrophotometer, Waltham, MA, USA) at 235 nm [14].

### 3.5. Molecular Identification of the Promising PHB-Producing Isolates

#### 3.5.1. DNA Extraction and Polymerase Chain Reaction (PCR) Amplification of 16S rRNA Gene

The bacterial genomic DNA of the promising isolates was extracted from 5 mL of the bacterial cultures grown overnight in broth medium, using a modified QIAamp DNA Mini Kit (Qiagen Inc., Valencia, CA, USA) [54]. The extracted DNA from each bacterial isolate was used as a template for the amplification of the 16S rRNA gene using the universal primers 5′ CCA GCA GCC GCG GTA ATA CG 3′ and 5′ATC GG(C/T) TAC CTT GTT ACG ACT TC 3′. An electrophoresis unit (30 min at 150 V) was used for the gel running and migration of the amplified genes. Using a gel documentation system, the migrated bands were seen under UV light. To verify the presence of properly sized amplicons, the PCR product for each isolate was compared against a 1-kb DNA ladder.

#### 3.5.2. Sequencing and GenBank Accession Numbers

A product of the right size was purified with a Taka R agarose gel DNA purification kit (version 2.0) (Qiagen Inc., Valencia, CA, USA), and sequenced in both directions using an ABI 3730 automated sequencer (Macrogen, Korea). The obtained sequence of the isolate was aligned and contrasted with the deposited sequences in GenBank [55]. To determine the taxonomic position of the isolate, a phylogenetic tree was built with MEGA version 5.10 program, Pennsylvania, USA, using a neighbor-joining algorithm. In addition, the Jukes-Cantor distance estimation strategy with bootstrap analyses for 1000 replicates was performed. The nucleotide sequences of the amplified 16S rRNA genes of the isolates were deposited in the GenBank nucleotide sequence databases.

### 3.6. Optimization of PHB Production

The fermentation process was performed in a 250 mL Erlenmeyer flask containing 50 mL of Zobell marine broth (pH = 7.5) in an incubated shaker (35 °C/ 150 rpm) for varying periods (24 h to 144 h). The effect of various factors on the PHB production were optimized; pH = 6.5–9.0, incubation temperature (25–45 °C), NaCl concentration (2–10% *w*/*v*), carbon sources (glucose, maltose, sucrose, arabinose, fructose, and date molasses), nitrogen sources (peptone, ammonium chloride, glycine, potassium nitrate, urea, yeast extract, and ammonium sulfate), and C:N (*w*/*w*) ratios of glucose and peptone (4.7:1, 9.2:1, 18.4:1, and 36.8:1). The cell dry weight (biomass) was determined by the collected cell pellets by centrifugation (6000 rpm/10 min), dried in an oven at 60 °C, and then expressed as g/L. All of the experiments were performed in triplicate.

### 3.7. Characterization of Extracted PHB

#### 3.7.1. Fourier Transform Infrared Spectroscopy (FTIR) Analysis

First, 1.0 mg of extracted PHB was dissolved in 5 mL of chloroform, and one drop of the mixture was added to a KBr disk. The spectrum was observed after the evaporation of the solvent under a vacuum at 400–4000 cm^−1^, using an IR double beam spectrophotometer (Shimandzu, Japan). The line-scan spectra were based on 32 scans and a resolution of 4 cm^−1^ [52].

#### 3.7.2. Nuclear Magnetic Resonance (NMR) Analysis

^1^H NMR and ^13^C NMR analyses were performed using an NMR 500 MHz Ultra Shield Bruker spectrophotometer (San Jose, CA, USA) and on an ECA-500 II Jeol spectrophotometer (JEOL Ltd., Tokyo, Japan). First, 5.0 mg of the extracted PHB was dissolved in deuterated chloroform (CDCl_3_), and the solution was analyzed at 500 MHz on a Bruker and Jeol spectrophotometer. Tetramethyl saline (TMS) was used as an external reference [21].

#### 3.7.3. Gas Chromatography-Mass Spectrometry (GC-MS) Analysis

For the methanolysis of the PHB, the samples were suspended in 1.0 mL chloroform and 1.0 mL H_2_SO_4_/methanol (15:85) in a screw-capped tube, and then heated to 100 °C for 2 h. After cooling, 0.5 mL of demineralized water was added, and the solution was vortexed for 1 min. The organic phase containing the resulting methyl esters’ monomers were identified by Varian Saturn 2100T GC-MS, equipped with a Saturn 2100 mass detector (Mundelein, IL, USA). The analysis was performed using a CP-SIL 8 CB column 30 m × 0.25 mm, with a 0.25 µm film thickness with a mobile phase of helium, and a flow rate of 1 mL/min. The fragmentation pattern of the obtained mass spectra was analyzed by NIST 98 mass library software, Gaithersburg, MD, USA [14,24].

### 3.8. Statistical Analysis

A one-way analysis of variance was carried out and all of the significance analyses were performed at *p* ≤ 0.05, using Minitab for Windows software (State College, PA, USA), package version 15. The error bars represent the standard error of the mean for *n* = 3.

## 4. Conclusions

The eco-friendly biodegradable plastic, PHB granules were accumulated by four marine bacteria obtained from mangrove rhizospheres. The maximum PHB production by the newly isolated *E. aquimaris* was obtained after 120 h, pH 8.0, 35 °C, and 2% NaCl, with glucose and peptone as the carbon and nitrogen sources, with a C:N ratio of 9.2:1. The characterization of the extracted polymer was conducted by FTIR, ^1^H NMR, ^13^C NMR, and GC-MS analysis to confirm its structure as a PHB. This study has provided valuable data about the optimized conditions for PHB production by marine *E. aquimaris*, which can make it a decent contender for numerous industrial applications as a substitution for petroleum-based plastics. Further studies must be conducted to obtained more economic production under fermenter conditions.

## Figures and Tables

**Table 1:** Summary of molecular identification of potential poly-β-hydroxybutyrate-producing marine bacteria.

Isolates ID	Isolates Name	Similarity of 16S rRNA Gene (%)	Genbank Accession Number
**KKU-MR35**	*Tamlana crocina*	99.69%	MH266206.1
**KKU-MR42**	*Bacillus aquimaris*	99.43%	MH266207.1
**KKU-MD13**	*Erythrobacter aquimaris*	99.39%	MH266210.1
**KKU-MD12**	*Halomonas halophila*	98.98%	MH266211.1
